# Algicidal Effects of a High-Efficiency Algicidal Bacterium *Shewanella* Y1 on the Toxic Bloom-Causing Dinoflagellate *Alexandrium pacificum*

**DOI:** 10.3390/md20040239

**Published:** 2022-03-30

**Authors:** Xi Chen, Dengyu Wang, Yanqun Wang, Pengfei Sun, Shuanghui Ma, Tiantian Chen

**Affiliations:** 1 College of Marine Life Science, Ocean University of China, Qingdao 266003, China; chenxi@ouc.edu.cn; 2 College of Environmental Science and Engineering, Ocean University of China, Qingdao 266100, China; wangdengyu@stu.ouc.edu.cn (D.W.); wangyq2019@stu.ouc.edu.cn (Y.W.); midnight459@163.com (S.M.); 3 Fourth Institute of Oceanography, Ministry of Natural Resources, Beihai 536000, China; sunpengfei@4io.org.cn; 4 Key Laboratory of Marine Ecological Environment, Ministry of Education, Ocean University of China, Qingdao 266100, China; 5 CAS Key Laboratory of Marine Ecology and Environmental Sciences, Institute of Oceanology, Chinese Academy of Sciences, Qingdao 266071, China

**Keywords:** algicidal bacteria, *Alexandrium pacificum*, algicidal effect, harmful algal blooms, *Shewanella*

## Abstract

*Alexandriumpacificum* is a typical toxic bloom-forming dinoflagellate, causing serious damage to aquatic ecosystems and human health. Many bacteria have been isolated, having algicidal effects on harmful algal species, while few algicidal bacteria have been found to be able to lyse *A. pacificum.* Herein, an algicidal bacterium, *Shewanella* Y1, with algicidal activity to the toxic dinoflagellate *A. pacificum*, was isolated from Jiaozhou Bay, China, and the physiological responses to oxidative stress in *A. pacificum* were further investigated to elucidate the mechanism involved in *Shewanella* Y1. Y1 exhibited a significant algicidal effect (86.64 ± 5.04% at 24 h) and algicidal activity in an indirect manner. The significant declines of the maximal photosynthetic efficiency (*F_v_*/*F_m_*), initial slope of the light limited region (alpha), and maximum relative photosynthetic electron transfer rate (rETRmax) indicated that the Y1 filtrate inhibited photosynthetic activities of *A. pacificum*. Impaired photosynthesis induced the overproduction of reactive oxygen species (ROS) and caused strong oxidative damage in *A. pacificum*, ultimately inducing cell death. These findings provide a better understanding of the biological basis of complex algicidal bacterium-harmful algae interactions, providing a potential source of bacterial agent to control harmful algal blooms.

## 1. Introduction

Over the last decades, harmful algal blooms (HABs) have increased in intensity and frequency and have turned into a serious ecological issue in coastal waters worldwide [1,2,3,4]. Toxic bloom-forming dinoflagellates can not only negatively influence their predators, including heterotrophic dinoflagellates [5], copepods [6], ciliates [7] and other zooplankton [8], but also have significantly negative impacts on co-occurring phytoplankton by releasing secondary metabolites as allelopathic chemicals [9]. *Alexandrium pacificum*, a typical toxic-HAB-causing dinoflagellate capable of discoloring seawater with sufficient cell densities, is commonly observed in estuarine and coastal waters around the world [10,11]. Frequent blooms of this species have been reported in the South China Sea, Ofunato Bay, Japan, Jinhae-Masan Bay, Korea, and Bizerte Lagoon, resulting in significant ecological damage and economic loss [12,13,14,15]. Furthermore, *A. pacificum* is a major producer of paralytic shellfish poisoning (PSP) [16]. Although PSP does not directly lead to the death of marine animals, it enters the human body through the food chain after being ingested by marine animals, and then poses a threat to human health and life safety [17,18].

In aquatic ecosystems, microalgae–bacteria interactions are complex. Many bloom-forming algal species have been known to form some associations with certain bacteria, but the reasons for these associations remain obscure [19]. The bacteria that closely associate with phytoplankton are believed to be involved in a wide range of interactions, including nutrient provision or competition [20,21,22], cell differentiation [23], and algicidal or bacterioprotective effects [24]. Previous studies have shown that algicidal bacteria have effective, species-specific, and environment-friendly features, which make them more outstanding than other chemical and physical methods in HABs control [25,26,27]. The ecological significance of algicidal bacteria was gradually recognized along with revelation of their specificity and diversity, stimulating attention towards the biological characteristics and ecological significance of these marine microorganisms.

Most known algicidal bacteria belong to the *Cytophaga*–*Flavobacteria*–*Bacteroidetes* (CFB) group or *Proteobacteria*, such as *Alteromonas*, *bacillus*, *Flavobacterium*, *Micrococcus*, *Pseudoalteromonas*, *Pseudomona,* and *Vibrio* [25,26,28]. Mechanisms of algicidal bacteria-lysing algal cells mainly take a few pathways: destruction of cell structure, alteration of enzymatic activities, and influence on algal photosynthesis or respiration [29,30]. Physiological and biochemical responses of algal cells were detected, including the decrease of chlorophyll *a*, interruption of the electron transport in photosystem II, decrease of the effective quantum yields, accumulation of excessive reactive oxygen species (ROS), inhibition of antioxidant enzymes activities, and increase of malodialdehyde (MDA) content [25,26,28]. Algicidal effects are regulated in the species of algicidal bacteria and target cells, and in a dose-dependent manner [26,28]. For *Alexandrium*, the general dosage is 1–5% (*v*/*v*), and the algicidal rate can reach more than 80% within 48 h [29,31]. Up to now, many algicidal bacteria have been identified to control *Alexandrium*, and the morphological structure, physiological and biochemical responses, and functional gene expression of algal cells after exposure to algicidal bacteria were analyzed to clarify their algicidal mechanism [29,31], but few studies have focused on algicidal bacteria against *A*. *pacificum*.

Herein, a highly effective algicidal bacterium, *Shewanella* Y1 (against *A. pacificum),* was isolated from Jiaozhou Bay and further confirmed with molecular characterization (*16S* rDNA sequencing). The objective of this study was to investigate its algicidal activity against the toxic dinoflagellate *A. pacificum*. By analyzing a series of changes of *A. pacificum*, we were able to identify the physiological characteristics of *A. pacificum* during the algicidal process. These findings will foster our understanding of complex algae-bacteria interactions and contribute to better understanding of the ecological roles of algicidal bacteria in the marine ecosystem.

## 2. Materials and Methods

### 2.1. Algal Cultures

*Alexandrium pacificum* (previously *Alexandrium catenella* strain ACHK-T, GenBank accession number KM091276) was kindly provided by Marine Planktology Laboratory, Ocean University of China, Qingdao, China. *A. pacificum* was maintained in f/2-Si medium [32] made with sterile natural seawater (salinity of 30 ± 0.1). The culture was incubated at 20 ± 1 °C under a 12:12 light:dark cycle, with cool white fluorescent light providing 75 μmol m^−2^ s^−1^. An antibiotic–antimycotic solution, with final concentrations of 100 μg mL^−1^ streptomycin, 0.05 μg mL^−1^ amphotericin B, and 100 I. U. penicillin (Solarbio Inc., Beijing, China), was added to the medium prior to inoculation to inhibit the growth of fungi and bacteria. This antibiotic mixture had no negative effects on the growth and survival of *A. pacificum*, as determined in preliminary experiments. The stock culture was maintained in the exponential growth phase by transferring into fresh f/2-Si medium bi-weekly.

### 2.2. Isolation, Purification and Identification of Algicidal Bacterium

Bacterial isolates were obtained from the Jiaozhou Bay, China (120.27° N, 36.10° E), using spread plate and streak plate isolation methods [33], and established as a culture library as described in Zheng et al. [34]. The bacterial strains were incubated at 25 °C (120 rpm for 72 h) in 2216E medium (5 g peptone, 1 g yeast extraction, 0.1 g ferric phosphorous acid dissolved in 1 L sterile seawater with a pH of 7.6–7.8) [35]. Bacterial isolates were frozen at −80 °C by adding liquid bacterial culture to a 50% freezing medium (65% glycerol, 0.1 M MgSO_4_ and 25 mM Tris–Cl, pH 8.0) [36].

Microbial genome DNA of the bacterial strain was extracted using the AxyPreP bacterial genome DNA kit (Axygen^®^, New York, NY, USA) according to the manufacturer’s instructions. The *16S* rDNA gene was amplified by polymerase chain reactions (PCR) using the universal primers 27F and 1492R (forward primer: 5′-AGAGTTTGATCMTGGCTCAG-3′, reverse primer: 5′-GATTACCTTGTTACGACTT-3′) [37]. PCR was conducted using a PCR Thermal Cycler (ABI-2720, Applied Biosystems, Waltham, MA, USA). The 50 μL PCR reaction mixture contained 1 μL (approximately 20 ng) of DNA template, 1.5 μL of each primer (10 μM), 25 μL of PCR mix (TaKaRa Bio Inc., Dalian, China), and 21 μL of de-ionized water. Thermocycling consisted of an initial denaturation at 95 °C for 5 min, followed by 35 cycles of 30 s at 95 °C, 30 s at 58 °C, and 1 min 30 s at 72 °C, and a final extension of 7 min at 72 °C. PCR-amplified product was confirmed by agarose gel electrophoresis (1%, *w*/*v*) stained with ethidium bromide and viewed under UV light.

**Figure 1:**
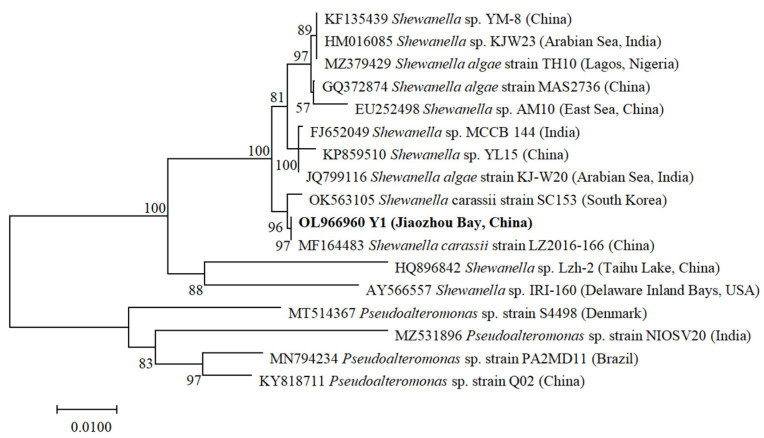
Molecular phylogenetic placement of the algicidal bacterial strain Y1 isolated from Jiaozhou Bay, China. The bold fonts are sequences from this study. Numbers at the nodes were bootstrapping support values larger than 50% after 1000 replicates in maximum likelihood (ML) analyses. The scale bar represents the number of substitutions per site.

The PCR product was purified respectively with a Takara MiniBest DNA fragment purification kit (Ver. 3.0) (Takara Bio Inc., Dalian, China) according to the manufacturer’s instructions, and sequenced bi-directionally by Shanghai Personalbio Technology Co., Ltd. Shanghai, China. For comparison and phylogenetic analysis, additional sequences were acquired from the National Center for Biotechnology Information (NCBI) database as indicated in Figure 1. Multiple alignments were performed using the Muscle algorithm in MacVector 13.5 (MacVector Inc., Apex, NC, USA). Sequences were trimmed to the same length. Maximum likelihood (ML) analysis was carried out using the MEGA 6 [38] with Tamura-Nei model of substitution and a gamma-shaped distribution of substitution rates among sites, which was determined through the Modeltest. All positions with gaps and missing data were eliminated. One-thousand bootstrap replicates were done to evaluate the robustness of the clades.

### 2.3. Algicidal Activity and Algicidal Mode Experiment

In order to investigate the algicidal activity and algicidal mode of strain Y1, the chlorophyll *a* (Chl *a*) content of *A. pacificum* was monitored. Bacterial strain Y1 was first inoculated into 50 mL 2216E medium for 24 h (25 °C, 120 rpm) to obtain the stationary bacterial culture, then the bacterial filtrate and the bacterial pellets were obtained by filtration (Millipore PC filters, 0.22-μm pore size) and centrifugation (7000 rpm, 5 min), respectively. The bacterial pellets were washed twice with sterile f/2-Si medium, and the pellets were resuspended in sterile f/2-Si medium. The algicidal activities were investigated by inoculating 150 mL of representative, exponentially growing *A. pacificum* culture (an initial density of approximately 10^4^ cells mL^−1^ without antibiotic–antimycotic solution addition) with 3 mL of the bacterial cultures, the bacterial filtrate or the washed bacterial pellets (final concentration of 2%, *v*/*v*). In addition, an equivalent volume of 2216E medium was added in the separate culture as experimental control. All treatments were conducted in triplicate and incubated under the same condition as mentioned above.

Chl *a* content was monitored at time points of 0.5 h, 1 h, 2 h, 4 h, 6 h, 12 h, 24 h, 36 h, and 48 h inoculation. Chl *a* was collected by filtering 10 mL of algal cultures through the Whatman GF/F filters (0.7-μm pore size, 25 mm in diameter, Whatman Inc., Beijing, China) with a vacuum pump (<0.04 MPa). Then the filters were wrapped against light in aluminum foils and stored at −20 °C for further processing. Chl *a* concentrations were measured using a fluorometric method after 24 h in 90% acetone in the dark at 4 °C [39] with a Turner Designs 10-005R fluorometer. The algicidal rate was calculated using the equation:

Algicidal rate (%) = (C_C_ − C_T_)/C_C_ × 100%

where C_C_ is the Chl *a* content in the treated algal cultures and C_T_ is the Chl *a* content in the control cultures.

### 2.4. Algicidal Effects of Y1 Filtrate on A. pacificum

Based on the above experiments, Y1 filtrate was used for further analysis as the potential active compound. Bath cultures of exponentially growing *A. pacificum* (700 mL) were prepared in 1 L culture flasks, with an initial density of approximately 10^4^ cells mL^−1^ without antibiotic–antimycotic solution addition. To investigate the response of *A. pacificum* to bacterial filtrate, *A. pacificum* cultures were inoculated with the bacterial filtrate (cultivated for 24 h, Millipore PC filters, 0.22-μm pore size) at a final concentration of 2% (*v*/*v*). In addition, an equivalent volume of sterile 2216E medium was added in the separate culture as experimental control. All treatments were conducted in triplicate and incubated under the same condition as mentioned above.

Morphological variation and algal abundance were assessed and further quantified at time points of 0 h, 6 h, 12 h, 24 h, 36 h and 48 h inoculation. Live observation of the algal culture (1 mL) was made at 400× magnification and recorded with an Olympus DP73 digital camera attached to an Olympus BX51 microscope. To determine algal abundance, subsamples (1 mL) were collected and fixed with acidic Lugol’s solution (final concentration 2%), then enumerated using triplicate plankton counting chambers under an Olympus BX51 microscope. The morphological distribution was calculated as the percentage of impaired cells when at least 100 cells per sample were counted. Photosynthesis activity, reactive oxygen species levels, lipid peroxidation, antioxidative enzyme, and antioxidant of algal cells were assessed at the four time points (0 h, 0.5 h, 1 h, 2 h, and 4 h) as described below.

### 2.5. Photosynthetic Activity of A. pacificum during the Algicidal Process

To assay the photosynthetic activity of *A. pacificum* exposed to the Y1 filtrate, the three photosynthetic parameters were analyzed. Aliquots of 2 mL algal culture were used to determine the chlorophyll fluorescence of *A. pacificum* using a FEM-2 pulse-modulated fluorometer (Hansatech, Norfolk, UK). The maximal photosynthetic efficiency (*F_v_*/*F_m_*) was calculated as:

*F_v_*/*F_m_* = (*F_m_* − *F_o_*)/*F_m_*

where *F_v_* represented the variable fluorescence, *F_m_* represented the maximum fluorescence, and *F_o_* represented the minimum fluorescence. Samples were dark-adapted for 30 min [40] before transfer to a cuvette for the measurement of *F_o_*, and after a saturating pulse (0.3 s; 2600 μmol photonsm^−2^ s^−1^), the measurement of *F_m_*. The alpha (α, initial slope of the light limited region) and maximum rETR (rETR_max_, maximum relative photosynthetic electron transfer rate) were also measured at the same time. The photosynthetic parameters of *F_v_*/*F_m_*, alpha and rETRmax represent the potential maximum photosynthetic capacity, the photosynthesis efficiency, and maximal electron transport rates, respectively. 

### 2.6. ROS Levels of A. pacificum during the Algicidal Process

To assess the alteration of reactive oxygen species (ROS) level of *A.pacificum* exposed to Y1 filtrate, a fluorescent probe, 2′,7′-dichlorofluorescin diacetate (DCFH-DA), was used to detect cellular ROS level. As DCFH-DA enters a cell and reacts with ROS, the probe molecule is converted into dichlorofluorescein (DCF) [41]. DCF fluorescence intensity could reflect ROS level of *A. pacificum*. Aliquots of 20 mL algal cultures were pipetted and concentrated at 4 °C via gentle centrifugation (4500 rpm, 10 min) to form pellets and remove the supernatant, then the pellets were washed twice with sterile 0.01M PBS (pH 7.2–7.4) (Solarbio Inc., Beijing, China). The DCFH-DA (10 mM) was added to the samples and the mixture was incubated at 37 °C in the dark for 40 min with gentle blending every 5 min. Then, the cells were collected via gentle centrifugation (4500 rpm, 10 min) and washed twice with 1 mL PBS. The pellets were re-suspended in 1 mL of PBS and acquired with a flow cytometer AccuriTM C6 Plus (BD Biosciences, Franklin Lakes, NJ, USA) equipped with two lasers (50-mW semiconductor tube laser and 30-mW diode laser) for excitation. Dead cells and non-algal particles were excluded from the analysis by gating on SSC-H/FL3-H, and at least 5000 intact cells were collected. Green fluorescence of DCF was revealed by an FL-1 detector (515–545 nm wavelength band, flow rate: 35 μL/s, run time: 3 min). The BD Accuri C6 Plus software (BD Biosciences, Franklin Lakes, NJ, USA) was used for acquisition and data analysis. 

### 2.7. Lipid Peroxidation, Antioxidative Enzyme and Antioxidant of A. pacificum during the Algicidal Process 

To evaluate the effect of strain Y1 on the physiological and biochemical responses of *A. pacificum*, the contents of superoxide dismutase (SOD), catalase (CAT), glutathione (GSH) and malondialdehyde (MDA) of *A. pacificum* were monitored. SOD, CAT and GSH are important antioxidant scavengers in cells, which were determined to investigate the cellular oxidative stress. MDA is used to reflect the degree of lipid peroxidation and is also an indicator of cellular oxidative damage [42]. Aliquots of 40 mL algal culture were pipetted and concentrated at 4 °C via gentle centrifugation (4500 rpm, 10 min) to form pellets and remove the supernatant. Then the pellets were washed twice with sterile 0.1 M PBS (pH 7.2–7.4) (Solarbio Inc., Beijing, China). The washed algae cells were re-suspended in sterile 0.1 M PBS and sonicated at below 4 °C (180 w, ultrasonic time: 1 s, rest time: 3 s), then observed by microscope until no intact individual cells was observed. The crude enzyme solution was obtained by centrifugation (12,000 rpm for 10 min at 4 °C). The processed crude enzyme solution was stored at −80 °C until further analysis. The alteration of MDA and GSH, and the activities of SOD and CAT, were assessed by using the relative kit (Nanjing Jiancheng Bioengineering Institute, Nanjing, China) according to the manufacturer’s instructions. 

### 2.8. Algicidal Activity of Y1 Filtrate toward Several HABs Species

The algicidal activity of Y1 filtrate was further detected in other algal species, including dinoflagellates (*Alexandrium tamarense*, *Prorocentrum donghaiense*, *Heterosigma akashiwo* and *Karenia mikimotoi*), and diatoms (*Skeletonema costatum* and *Chaetoceros curvisetus*). Vials (in triplicate) containing 150 mL of each species in exponential growth phases were inoculated with Y1 filtrate at a final concentration of 2% (*v*/*v*). In addition, an equivalent volume of sterile 2216E medium was added in the separate culture as experimental control. The Chl *a* content was monitored at 24 h after Y1 filtrate-added, and the algicidal rate was estimated as described in Section 2.3. All treatments were conducted in triplicate and incubated under the same condition as mentioned above.

### 2.9. Statistical Analysis

All data were presented as mean ± standard error of the mean (SE) in the text and subjected to a one-way analysis of variance (ANOVA) by Duncan’s post hoc multiple comparison test using the SPSS 25.0 for Windows (SPSS, Chicago, IL, USA), with the significance level of *p* < 0.05.

## 3. Results

### 3.1. Identification of Algicidal Bacterium

A total of 15 bacterial isolates were obtained from Jiaozhou Bay, China, of which 4 strains were found to exert algicidal activity against *A. pacificum*. Among the 4 strains, Y1 had an apparent negative effect on the growth of *A. pacificum* and was used for further investigation.

A unique amplicon, with 1455 bp in length after trimming the primers and ambiguous sites at both ends (GenBank accession number OL966960), was obtained from the bacterial isolate Y1 from Jiaozhou Bay, China. The bacterial isolate Y1 was identified by *16S* rDNA sequencing to belong to the Class γ-p, genus *Shewanella*, and was 97% similar to a *Shewanella carassii* LZ2016-166 isolated from sediments of China. All the *Shewanella* sequences joined together forming a monophyletic clade with 100% bootstrapping support and formed two sister sub-clades. One well-defined sub-clade included sequences from India, Nigeria, South Korea, and China, and the other comprised those sequences from USA and China (Figure 1). Two *Shewanella* strains, *Shewanella* IRI-160 and *Shewanella* Lzh-2, were proven to possess algicidal activity [43,44]. Strain Y1 and strain IRI-160/Lzh-2 separately developed into independent branches, indicating *Shewanella* Y1 was not same strain as *Shewanella* IRI-160/Lzh-2.

### 3.2. Algicidal Mode of Algicidal Strain Y1

**Figure 2:**
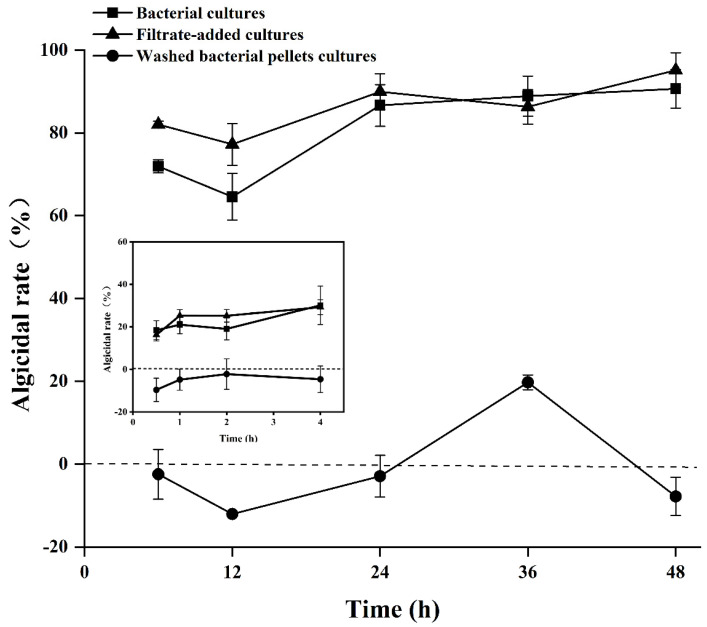
Algicidal modes of strain Y1 on *A. pacificum*. The inset figure represents algicidal rate at 0.5–4 h inoculation. Bacterial cultures indicate algal cultures with 2% (*v*/*v*) Y1 cultures addition; filtrate-added cultures indicate algal cultures with 2% (*v*/*v*) Y1 filtrate addition. Washed bacterial pellets cultures indicate algal cultures with 2% (*v*/*v*) washed bacterial pellets cultures added. Experiments were performed in triplicate. Error bars indicate the standard error.

For the bacterial cultures and filtrate-added cultures, the algicidal rates showed a similar pattern of variation, and no significant difference was observed between the two treatments (*p* > 0.05). The algicidal rates of the bacterial cultures and filtrate-added cultures began to increase from 0.5 h inoculation and reached 30.14 ± 9.05% and 29.24 ± 3.49% at 4 h respectively. After 24 h inoculation, the algicidal rates of the bacterial cultures and filtrate-added cultures reached a stable level, lysing 86.64 ± 5.04% and 89.95 ± 4.35% of the algal cells respectively, then increased to 90.66 ± 4.65% and 95.14 ± 4.24% at 48 h (Figure 2). In the washed bacterial pellets cultures, the algicidal rate exhibited a significant difference from the bacterial cultures and filtrate-added cultures (*p* < 0.05), which ranged from −12 ± 0.04% to 19.74 ± 1.77% (Figure 2). These findings suggested strain Y1 secreted algicidal substances to attack *A. pacificum*, thus bacterial filtrate was used for further analysis as the potential active compound.

### 3.3. Effects of Y1 Filtrate on A. pacificum 

**Figure 3:**
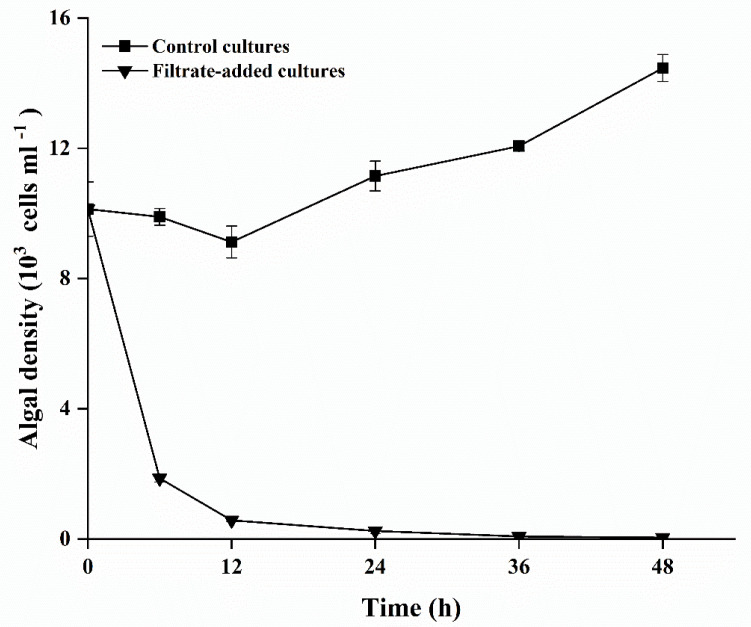
The growth curves of *A. pacificum* after exposure to Y1 filtrate. Control cultures indicate algal cultures with 2% (*v*/*v*) 2216E medium addition; filtrate-added cultures indicate algal cultures with 2% (*v*/*v*) Y1 filtrate addition. Experiments were performed in triplicate. Error bars indicate the standard error.

In the control cultures, *A. pacificum* showed steady growth during the experiment, reaching 1.45 ± 0.01 × 10^4^ cells mL^−1^ by 48 h. In the bacterial filtrate-added cultures, the growth of *A. pacificum* was significantly affected by introduction of Y1 filtrate. The cell density of *A. pacificum* decreased dramatically after 6 h inoculation, and the final density was 46 ± 12 cells mL^−1^ (Figure 3).

**Figure 4:**
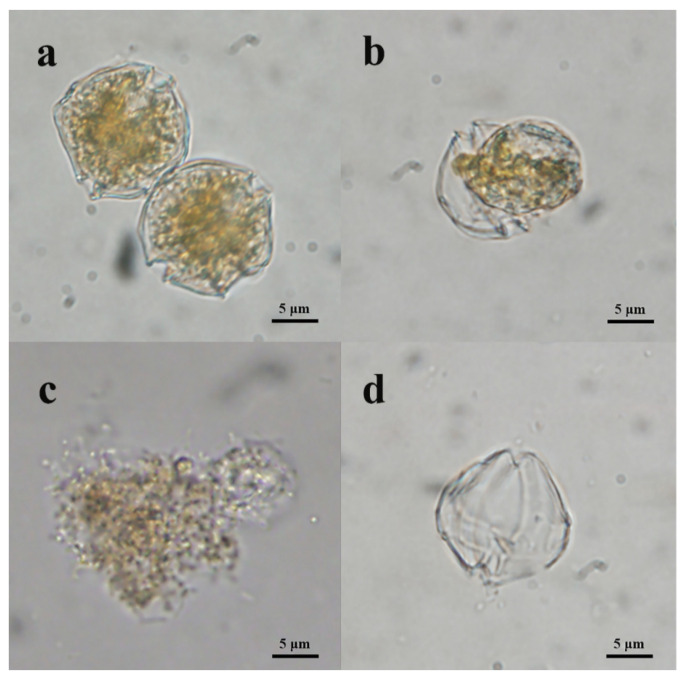
Morphological changes in *A. pacificum* after exposure to Y1 filtrate (2%, *v*/*v*) under an optical microscope (10 × 40). (**a**) Control with the addition of the same volume of 2216E; (**b**) 6 h treatment with Y1 filtrate; (**c**) 12 h treatment with Y1 filtrate; (**d**) 24 h treatment with Y1 filtrate.

**Figure 5:**
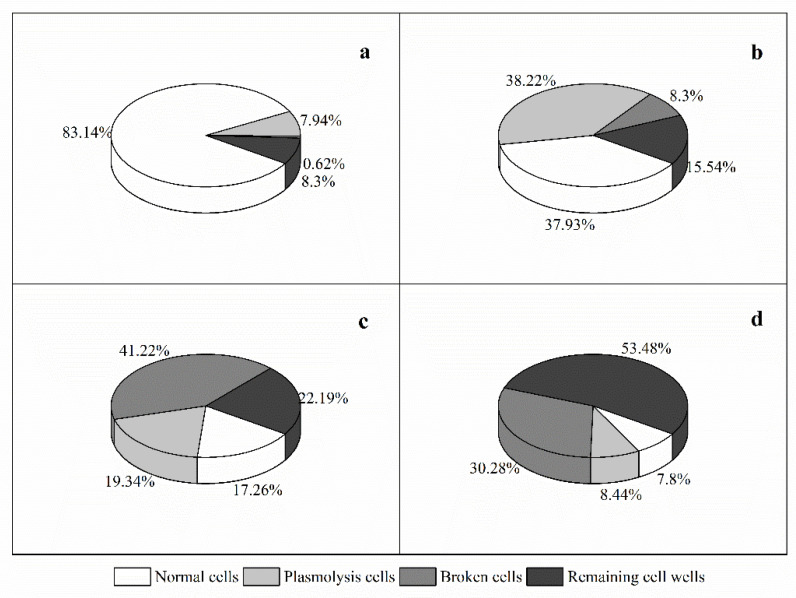
The distribution of the different morphologies of *A. pacificum* after exposure to Y1 filtrate (2%, *v*/*v*) under an optical microscope. (**a**) Control with the addition of the same volume of 2216E; (**b**) 6 h treatment with Y1 filtrate; (**c**) 12 h treatment with Y1 filtrate; (**d**) 24 h treatment with Y1 filtrate.

Compared with the control cultures, the bacterial filtrate-added cultures exhibited significant morphological differences and even structural damage. The normal *A. pacificum* cell was brownish yellow and swam fast. The cellular structures were intact with two distinct carapaces and dense cytoplasm (Figure 4a). However, the structural integrity of the algal cells was lost, and plasmolysis and vacuolization, and even degradation, of the cytoplasm were observed in the treated cells after 6 h (Figure 4b), and the percentage of plasmolysis cells was 38.22 ± 3.73 (Figure 5b). With extended exposure (12 h), the cytoplasm became completely divorced from the cell well, and began to degrade into small, irregular debris particles (Figure 4c), with the percentage of broken cells at 41.22 ± 0.1 (Figure 5c). At 24 h, the algal cell was thoroughly disintegrated, and only the remaining cell well was observed in the culture (Figure 4d), and the percentage of remaining cell walls was 53.48 ± 3.37 (Figure 5d).

### 3.4. Variation of Photosynthetic Activity of A. pacificum under the Action of Y1 Filtrate

**Figure 6:**
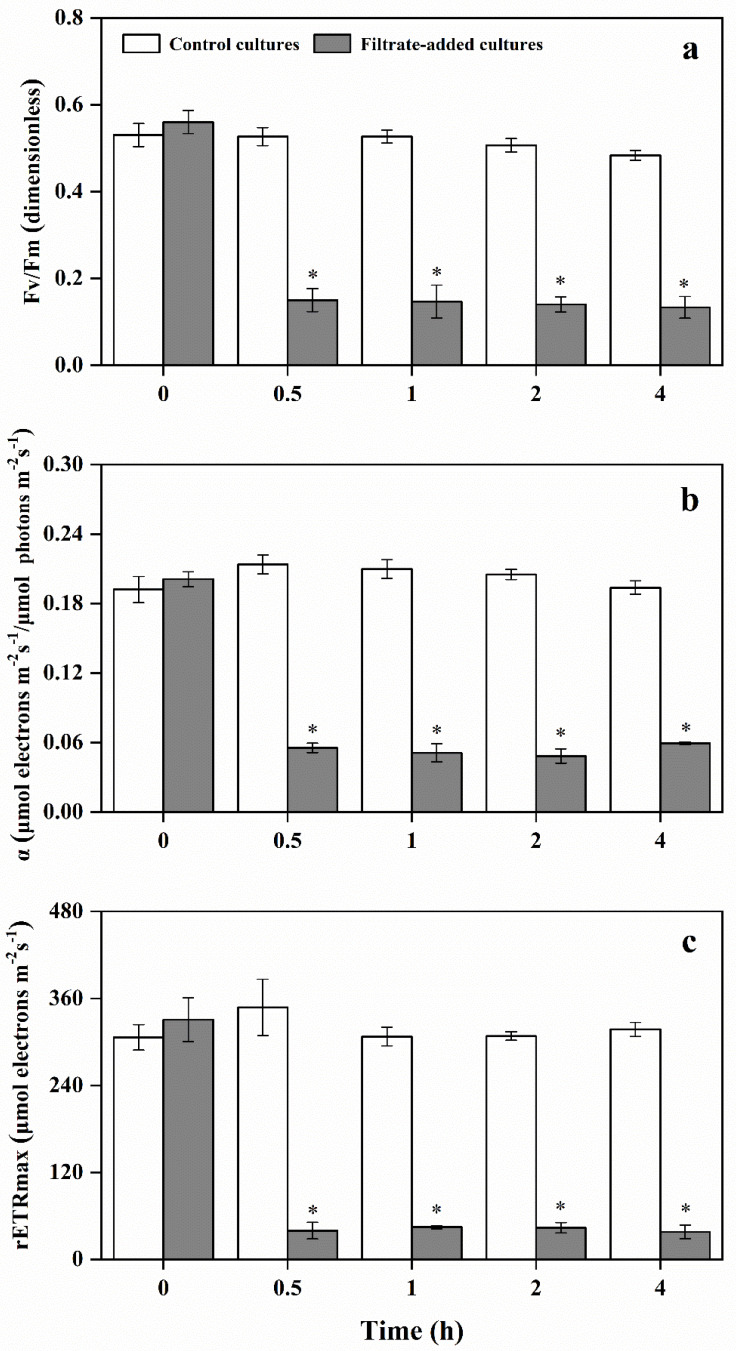
Effects of Y1 filtrate on photosynthetic activity of *A. pacificum*. (**a**) maximal photosynthetic efficiency (*F_v_*/*F_m_*); (**b**) initial slope of the light limited region (alpha); (**c**) maximum relative photosynthetic electron transfer rate (rETRmax). Control cultures indicate algal cultures with 2% (*v*/*v*) 2216E medium addition; filtrate-added cultures indicate algal cultures with 2% (*v*/*v*) Y1 filtrate addition. Experiments were performed in triplicate. Error bars indicate the standard error. Statistical significances across control at the same time of sampling are indicated with an asterisk (*p* < 0.05).

Photosynthesis is an important determinant of the ability of marine microalgae to survive biotic stresses, and the *F_v_*/*F_m_* is widely used to evaluate photosynthetic activity of microalgae. The photosynthetic activities of *A. pacificum* were overtly impacted by Y1 filtrate (Figure 6a). The *F_v_*/*F_m_* of *A. pacificum* remained steady in the control cultures, with near-optimal quantum yield values (*F_v_*/*F_m_* = 0.5) over the experimental period. *F_v_*/*F_m_* values in the filtrate-added cultures ranged from 0.56 ± 0.03 to 0.13 ± 0.03, significantly lower than that of the control cultures (0.53 ± 0.03 to 0.48 ± 0.01, *p* < 0.05).

Alpha (α) is one of the key photosynthetic parameters indicating photosynthetic activity. In the control cultures, alpha value was maintained at 0.20 ± 0.01. Alpha value in the filtrate-added cultures decreased to 0.05 ± 0.01 by 2 h, which was significantly lower than that of the control cultures over the experimental period (*p* < 0.05) (Figure 6b). 

The maximum relative electron transfer rate (rETR_max_) of *A. pacificum* showed a similar pattern to *F_v_*/*F_m_* and the alpha (Figure 6c). In the control cultures, the ETR_max_ value ranged from 306.47 ± 17.51 to 347.8 ± 38.91, while the ETR_max_ value significantly decreased in the filtrate-added cultures after 0.5 h inoculation (*p* < 0.05), which ranged from 37.83 ± 9.42 to 330.73 ± 30.02.

### 3.5. Physiological and Biochemical Responses of A. pacificum under the Action of Y1 Filtrate

**Figure 7:**
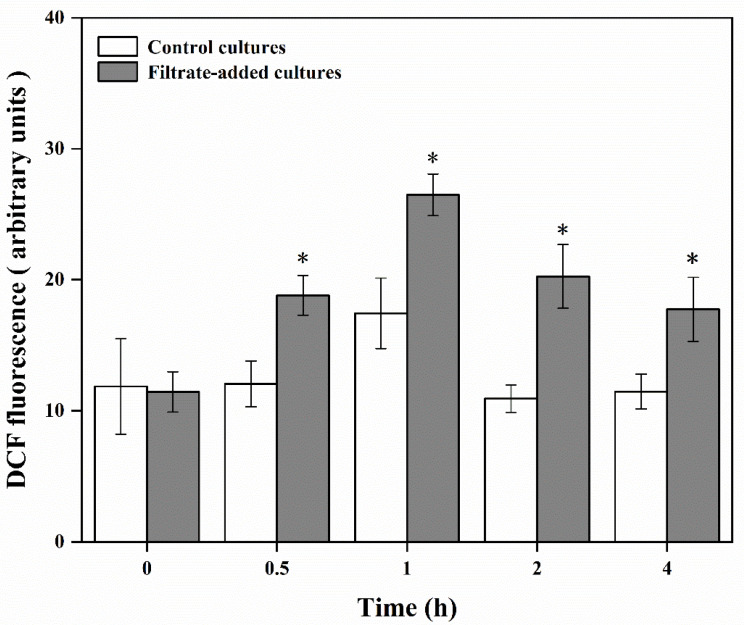
Effects of Y1 filtrate on reactive oxygen species (ROS) of *A. pacificum*. Control cultures indicate algal cultures with 2% (*v*/*v*) 2216E medium addition; filtrate-added cultures indicate algal cultures with 2% (*v*/*v*) Y1 filtrate addition. Experiments were performed in triplicate. Error bars indicate the standard error. Statistical significances across control at the same time of sampling are indicated with an asterisk (*p* < 0.05).

In the control cultures, the ROS level increased briefly at 1 h, reaching the peak (17.44 ± 2.67). Beyond that, the ROS level was maintained at 11.58 ± 0.49. Compared with the control cultures, the ROS level was significantly increased in the filtrate-added cultures (*p* < 0.05), which ranged from 11.44 ± 1.54 to 26.48 ± 1.59. The ROS levels of 0.5, 1, 2, 4 h in the filtrate-added cultures were 1.56-fold, 1.52-fold, 1.85-fold, and 1.55-fold those of the control cultures, respectively (Figure 7).

**Figure 8:**
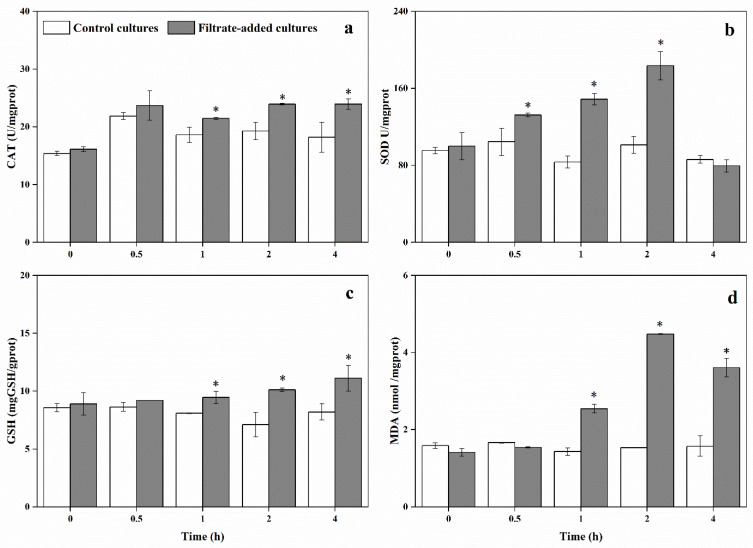
Effects of Y1 filtrate on the activity of catalase (CAT) (**a**) and superoxide dismutase (SOD) (**b**), and the content of glutathione (GSH) (**c**) and malondialdehyde (MDA) (**d**) of *A. pacificum*. Control cultures indicate algal cultures with 2% (*v*/*v*) 2216E medium addition; filtrate-added cultures indicate algal cultures with 2% (*v*/*v*) Y1 filtrate addition. Experiments were performed in triplicate. Error bars indicate the standard error. Statistical significances across control at the same time of sampling are indicated with an asterisk (*p* < 0.05).

In the control cultures, the CAT activity of *A. pacificum* remained steady (18.67 ± 2.31 U/mgprot) throughout the experiment (Figure 8a). Within first 0.5 h inoculation, no significant difference was observed between the two treatments (*p* > 0.05). With longer exposure time, the CAT activity of *A. pacificum* in the filtrate-added cultures increased continuously, peaking at 4 h (23.9 ± 0.92 U/mgprot). The CAT activities of 1, 2, 4 h treatment with filtrate were 1.15-fold, 1.24-fold, and 1.31-fold those of the control cultures, respectively.

The SOD activities of *A. pacificum* were overtly impacted by Y1 filtrate (Figure 8b). The SOD activity in the filtrate-added cultures ranged from 79.4 ± 6.4 to 183.44 ± 14.89, which was a significant difference from that of the control cultures (83.38 ± 6.28 to 104.5 ± 14.14 U/mgprot). At 2 h inoculation, SOD activity in the filtrate-added cultures reached the peak (188.44 ± 14.89 U/mgprot), which was 1.8-fold that of the control cultures, then sharply decreased to 79.4 ± 6.4 U/mgprot, while no significant difference was observed in the SOD activities between the two groups after 2 h inoculation (*p* > 0.05).

The GSH content showed similar pattern to CAT activity (Figure 8c). There was a significant difference between the two cultures after 0.5 h inoculation (*p* < 0.05). The GSH content remained steady in the control cultures, ranging from 7.1 ± 1.06 to 8.62 ± 0.37 mgGSH/gprot over the whole experimental period. The contents of GSH in the filtrate-added cultures began to increase after 0.5 h and the maximum values (11.11 ± 1.11 mgGSH/gprot) were 1.36-fold that of the control cultures at 4 h inoculation (*p* < 0.05).

In the control cultures, MDA value ranged from 1.44 ± 0.09 to 1.66 ± 0.01 nmol/mgprot. While MDA value significantly increased in the filtrate-added cultures after 1 h inoculation (*p* < 0.05), which ranged from 2.54 ± 0.11 to 3.61 ± 0.24 nmol/mgprot. In the filtrate-added cultures, the MDA content reached maximum value at 2 h inoculation, which was 2.9-fold that of control cultures. Although the MDA contents in filtrate-added cultures declined slightly after 2 h, the content was still much higher than that of the control cultures (Figure 8d).

### 3.6. Algicidal Activity of Y1 Filtrate toward Several HABs Species

**Figure 9:**
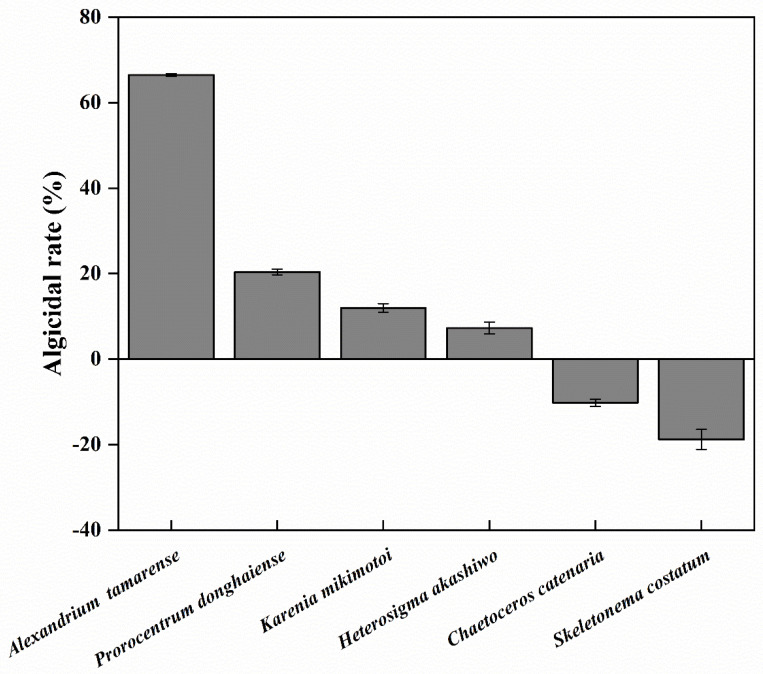
The algicidal activity of Y1 filtrate against several HABs species after 24 h co-incubation. Error bars indicate the standard error.

Various effects were observed among the six tested species (Figure 9). Within 24 h incubation, Y1 filtrate showed the strongest algicidal activity on *A. tamarense* (66.45 ± 0.3%), a slight negative effect on *Prorocentrum donghaiense* (20.36 ± 0.65%), *Karenia mikimotoi* (11.96 ± 1.03%), and *Heterosigma akashiwo* (7.27 ± 1.37%), a slight stimulatory effects on the growth of diatoms, *Chaetoceros catenaria* (−10.23 ± 0.83%) and *Skeletonema costatum* (−18.82 ± 2.33%).

## 4. Discussion

Algicidal bacteria have gained growing attention since discovery of their advantageous low cost and species-specific features for controlling harmful algal blooms [25,26,27]. However, the interactions between algicidal bacteria and the toxic bloom-causing dinoflagellates have received limited attention, especially for the relationship between *A. pacificum* and algicidal bacteria. In this study, a high-efficiency algicidal strain *Shewanella* Y1 (against *A. pacificum*) was isolated from Jiaozhou Bay, China. Our results demonstrated that the *Shewanella* Y1 indirectly exerted a strong algicidal effect on *A. pacificum* by secreting extracellular algicidal substance. The Y1 filtrate caused a series of changes of *A. pacificum*, including decreased cell density, cellular degradation, impaired physiological activities, and strong oxidative damage. These results will foster our understanding of bacterium–protist interactions in the marine ecosystem, which will be helpful for estimating the ecological role of this algicidal bacterium and its potential use as a microbial control agent for HABs.

The known algicidal bacteria against *A. tamarense* or *A. pacificum* (previously *Alexandrium catenella*) are mainly *Vibrio*, *Pseudoalteromonas*, *Flavobacterium*, *Joostella*, *Deinococcus*, *Brevibacterium*, and *Bacillus* (Table 1). Isolate *Shewanella* IRI-160 was obtained from the inlet of Indian River Bay, Delaware, which was the first reported bacterium of the genus *Shewanella* to have algicidal effects on phytoplankton. *Shewanella* IRI-160 exerted strong algae-killing effects on naked dinoflagellates (algicidal rate more than 85%, 48 h), such as *Karlodinium veneficum*, *Karenia brevis* and *Gyrodinium instriatum*, but the effects on thecate dinoflagellates, such as *Prorocentrum minimum*, *Oxyrrhis marina*, and *A. tamarense* were insignificant (algicidal rate less than 35%, 48 h) [43,45]. *Shewanella* Y1 displayed strong algicidal activity against *A. pacificum* and *A. tamarense* in the present study. Although the algicidal effect of IRI-160 filtrate was not evaluated for the thecate dinoflagellate, *A. pacificum*, it can be reasonably inferred that the algicidal effect of IRI-160 filtrate on *A. pacificum* is inefficent based on the different algicidal trends of *Shewanella* IRI-160 between the naked dinoflagellates and armored dinoflagellates. Future studies will test whether this assumption is true. Therefore, *Shewanella* Y1 is the first record of a *Shewanella* bacterium being algicidal to the harmful dinoflagellate *A. pacificum*. 

Y1 strain showed a higher algicidal rate on *A. pacificum* than other tested algal species in the present study, indicating that *A. pacificum* was a preferred target. Different strains of algicidal bacteria indicated various degrees of species specificity ranging from highly species-specific [54], to broadly specific [31]. The algicidal activity of Y1 filtrate against thecate dinoflagellate (*A. pacificum* or *A. tamarense*) was higher than that of naked dinoflagellate (*Karenia mikimotoi*), which is in agreement with a previous study [55]. *Alteromonas* FDHY-03 was proven to lyse *Prorocentrum donghaiense* (>90% algicidal rate in 24 h) by secreting an algicidal compound, glycosidase [55]. However, for *P. donghaiense*, *Shewanella* Y1 only showed 20.36 ± 0.65% algicidal rate in 24 h. Thus, it can be ruled out that the glucosidase played a major role in *Shewanella* Y1’s exertion of algicidal effects on target algae. Although there appears to be no clear mode of prey specificity for algicidal bacteria based on various studies [26], bacteria-produced algicidal substances could be a candidate for the selective inhibition of HABs.

Adding only the filtrate or exudate from the bacterial culture was used to distinguish between direct or indirect attack. Our algicidal mode experiment showed that the Y1 filtrate had algicidal activity similar to that of the Y1 cultures, but the washed bacterial pellets cultures showed dramatically lower algicidal activity, suggesting that strain Y1 indirectly attacks *A. pacificum* by secreting extracellular algicidal substances. Various algicidal strains have been classified based on their algicidal modes; while approximately 70% of the strains exhibited indirect algicidal attacks, the remaining exhibited direct algicidal attacks [27,28]. It is worth noting that individual strains showed both direct and indirect algicidal attacks [45,56]. Current data indicate that *Proteobacteria* are typically involved in indirect attacks, even though there is no clear link between bacterial class and algicidal mode [26,28]. The evident algicidal activity in the filtrate suggested that these strains could suppress algal cells via an indirect attack by releasing extracellular algicidal substances such as proteins, enzymes, or biological surfactants [57,58,59,60]. However, the number of identified algicidal substances is remarkably lower than that of reported algicidal bacteria, which may be attributed to a limitation in the separation and purification methods and identification technology for algicidal compounds.

In the filtrate-added cultures, the three key photosynthetic parameters showed a significant decrease compared with that of control cultures over the experimental period, suggesting that the photosynthetic activities were significantly suppressed. Impaired photosynthesis could block the electron transport, which would promote electron spillage and cause a significant increase in ROS production [61,62]. The elevated ROS in algal cells could cause significant dysfunction of physiological and metabolic processes, which could damage the crucial biomacromolecules including DNA, lipids, and proteins [42]. The ROS level in filtrate-added cultures was significantly higher than that of control cultures. The CAT and SOD activities and GSH contents were significantly enhanced at 1 h inoculation, suggesting that CAT, SOD, and GSH were activated to resist oxidative stress. Under the self-resistance of algal cells, the ROS level began to decrease after 2 h, but the effect was not completely alleviated. The MDA contents of *A. pacificum* after exposure to Y1 filtrate increased significantly in a short time, resulting in oxidative damage of membrane system (including plasma membrane and thylakoid membrane) and death of algal cells. These results suggested that Y1 filtrate could inhibit photosynthetic activities, induce the production of a large amount of ROS, cause oxidative damage to the membrane system, and eventually lead to cell rupture and death. 

## 5. Conclusions

In conclusion, *Shewanella* Y1 isolated from Jiaozhou Bay, China exerted a strong algicidal effect on *Alexandrium pacificum*, and algicidal activity in an indirect manner. Y1 filtrate altered physiological state, decreased cell density, inhibited photosynthetic activities, and caused strong oxidative damage in *A. pacificum*, ultimately inducing cell death. These findings highlight the fundamental cellular processes involved in the algicidal effects of *Shewanella* Y1 toward targeted algae collapsing. Further research on the discovery of algicidal compounds from *Shewanella* Y1 and their effect on harmful algal species should decode these complex algicidal mechanisms, which will enrich the species variety of algacidal bacteria and aid in developing strategies for controlling HABs.

## Figures and Tables

**Table 1:** The isolated algicidal bacteria associated with *A. pacificum* or *A. tamarense*.

Phylum	Genus	Killed Algae	References
*Proteobacteria*	*Vibrio* sp.	*A. tamarense*	[27,46]
		*A. catenella*	[47]
	*Pseudoalteromonas* sp.	*A. catenella*	[48]
	*Shewanella* sp.	*A. pacificum*	This study
*Bacteroidetes*	*Joostella* sp.	*A. tamarense*	[49]
	*Flavobacterium* sp.	*A. tamarense*	[50]
*Thermus*	*Deinococcus* sp.	*A. tamarense*	[29]
*Actinobacteria*	*Brevibacterium* sp.	*A. tamarense*	[51]
		*A. catenella*	[52]
*Firmicules*	*Bacillus* sp.	*A. catenella*	[53]

## Data Availability

The datasets generated and analyzed during the current study are available from the corresponding author on reasonable request.
